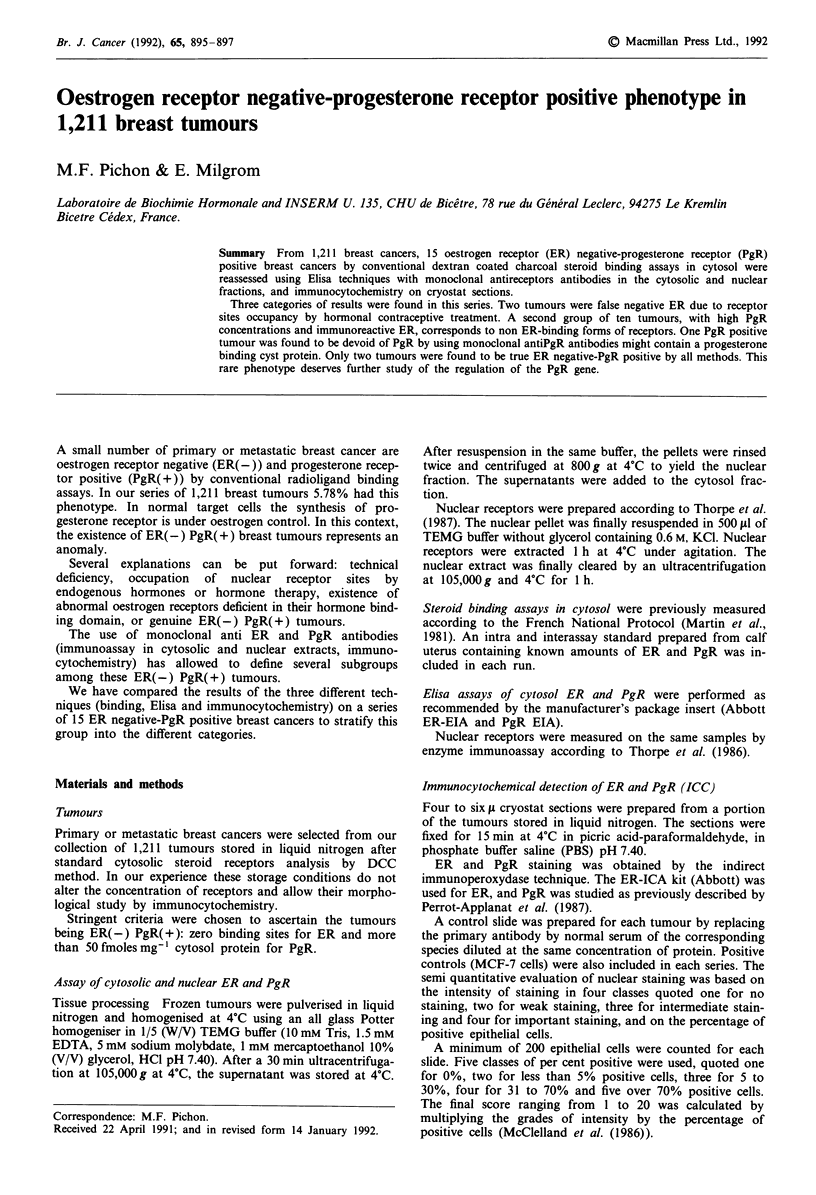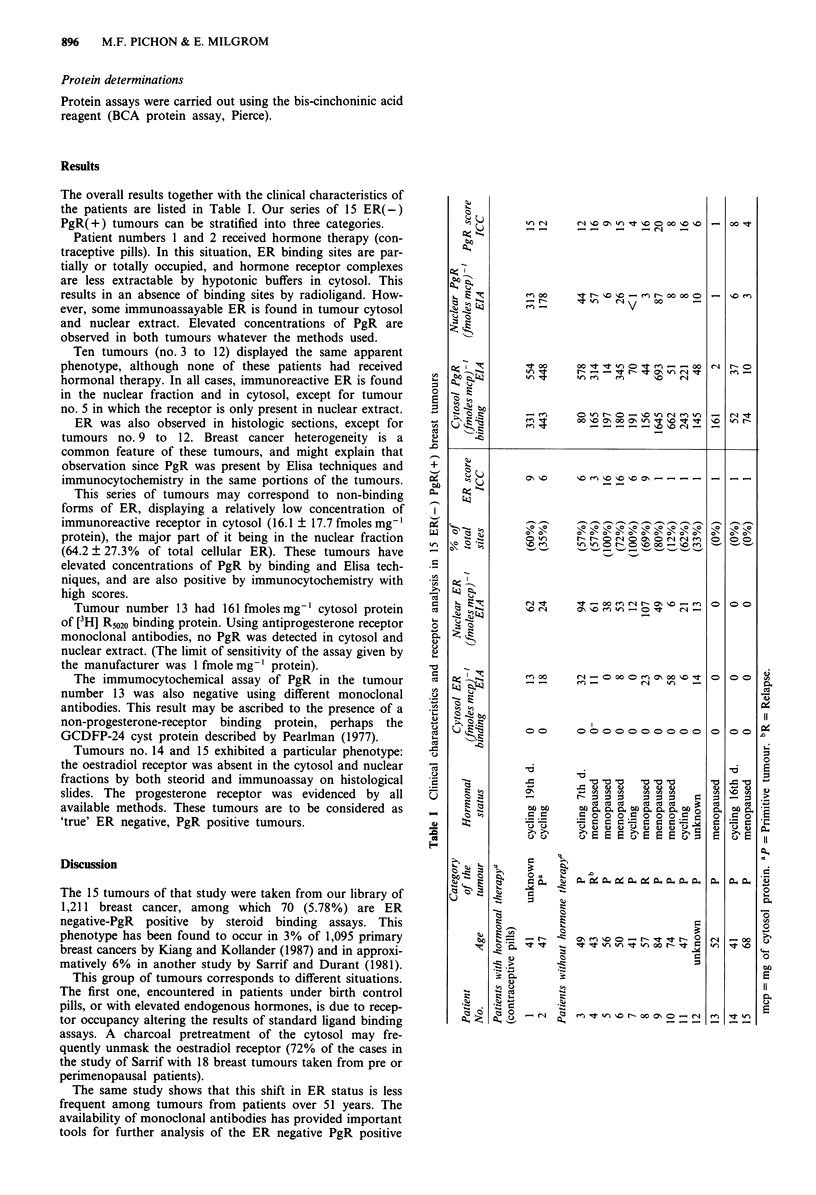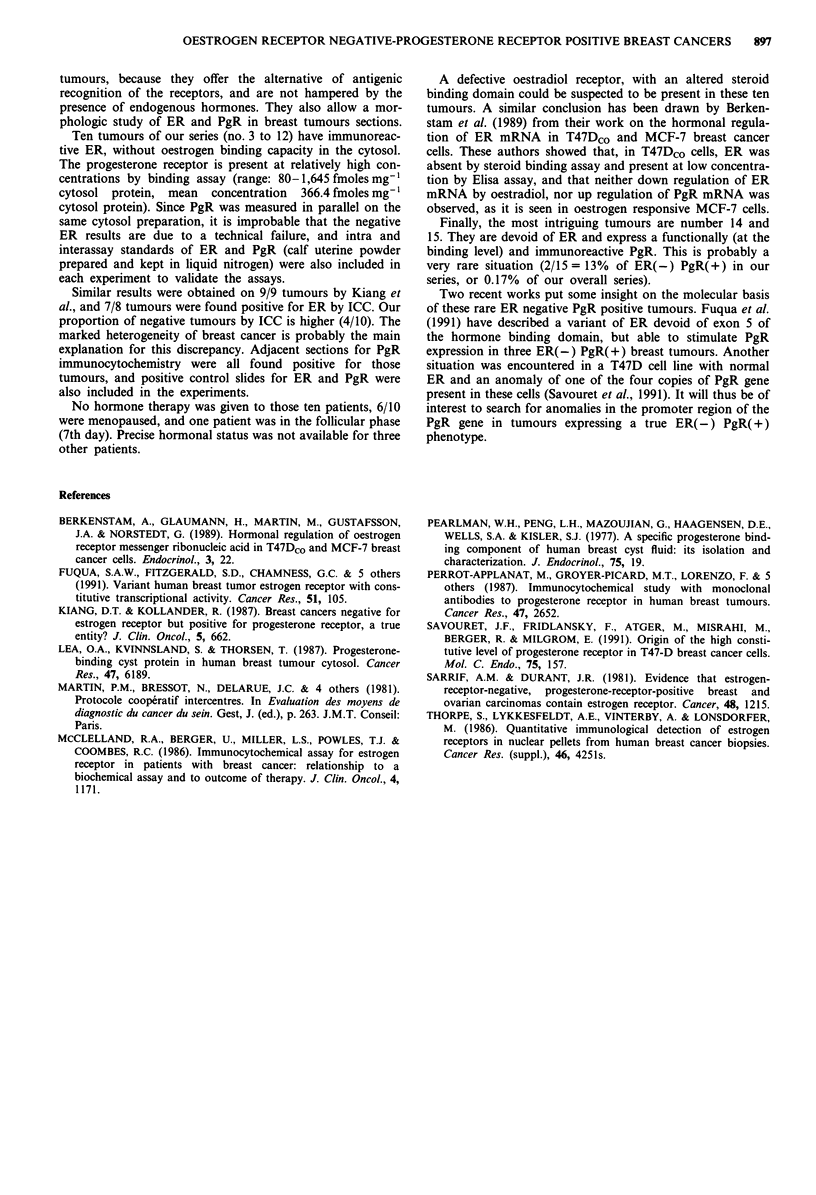# Oestrogen receptor negative-progesterone receptor positive phenotype in 1,211 breast tumours.

**DOI:** 10.1038/bjc.1992.187

**Published:** 1992-06

**Authors:** M. F. Pichon, E. Milgrom

**Affiliations:** Laboratoire de Biochimie Hormonale, CHU de Bicêtre, Le Kremlin Bicetre, France.

## Abstract

From 1,211 breast cancers, 15 oestrogen receptor (ER) negative-progesterone receptor (PgR) positive breast cancers by conventional dextran coated charcoal steroid binding assays in cytosol were reassessed using Elisa techniques with monoclonal antireceptors antibodies in the cytosolic and nuclear fractions, and immunocytochemistry on cryostat sections. Three categories of results were found in this series. Two tumours were false negative ER due to receptor sites occupancy by hormonal contraceptive treatment. A second group of ten tumours, with high PgR concentrations and immunoreactive ER, corresponds to non ER-binding forms of receptors. One PgR positive tumour was found to be devoid of PgR by using monoclonal antiPgR antibodies might contain a progesterone binding cyst protein. Only two tumours were found to be true ER negative-PgR positive by all methods. This rare phenotype deserves further study of the regulation of the PgR gene.


Br. J. Cancer (1992), 65, 895–897 © Macmillan Press Ltd., 1992

### Oestrogen receptor negative-progesterone receptor positive phenotype in 1,211 breast tumours

M.F. Pichon & E. Milgrom

*Laboratoire de Biochimie Hormonale and INSERM U. 135, CHU de Bicêtre, 78 rue du Général Leclerc, 94275 Le Kremlin Bicetre Cédex, France.*

**Summary** From 1,211 breast cancers, 15 oestrogen receptor (ER) negative-progesterone receptor (PgR) positive breast cancers by conventional dextran coated charcoal steroid binding assays in cytosol were reassessed using Elisa techniques with monoclonal antireceptors antibodies in the cytosolic and nuclear fractions, and immunocytochemistry on cryostat sections.

Three categories of results were found in this series. Two tumours were false negative ER due to receptor sites occupancy by hormonal contraceptive treatment. A second group of ten tumours, with high PgR concentrations and immunoreactive ER, corresponds to non ER-binding forms of receptors. One PgR positive tumour was found to be devoid of PgR by using monoclonal antiPgR antibodies might contain a progesterone binding cyst protein. Only two tumours were found to be true ER negative-PgR positive by all methods. This rare phenotype deserves further study of the regulation of the PgR gene.

A small number of primary or metastatic breast cancer are oestrogen receptor negative (ER(−)) and progesterone receptor positive (PgR(+)) by conventional radioligand binding assays. In our series of 1,211 breast tumours 5.78% had this phenotype. In normal target cells the synthesis of progesterone receptor is under oestrogen control. In this context, the existence of ER(−) PgR(+) breast tumours represents an anomaly.

Several explanations can be put forward: technical deficiency, occupation of nuclear receptor sites by endogenous hormones or hormone therapy, existence of abnormal oestrogen receptors deficient in their hormone binding domain, or genuine ER(−) PgR(+) tumours.

The use of monoclonal anti ER and PgR antibodies (immunoassay in cytosolic and nuclear extracts, immunocytochemistry) has allowed to define several subgroups among these ER(−) PgR(+) tumours.

We have compared the results of the three different techniques (binding, Elisa and immunocytochemistry) on a series of 15 ER negative-PgR positive breast cancers to stratify this group into the different categories.

### Materials and methods

### *Tumours*

Primary or metastatic breast cancers were selected from our collection of 1,211 tumours stored in liquid nitrogen after standard cytosolic steroid receptors analysis by DCC method. In our experience these storage conditions do not alter the concentration of receptors and allow their morphological study by immunocytochemistry.

Stringent criteria were chosen to ascertain the tumours being ER(−) PgR(+): zero binding sites for ER and more than 50 fmoles $mg^{-1}$ cytosol protein for PgR.

### *Assay of cytosolic and nuclear ER and PgR*

Tissue processing Frozen tumours were pulverised in liquid nitrogen and homogenised at 4°C using an all glass Potter homogeniser in 1/5 (W/V) TEMG buffer (10 mM Tris, 1.5 mM EDTA, 5 mM sodium molybdate, 1 mM mercaptoethanol 10% (V/V) glycerol, HCl pH 7.40). After a 30 min ultracentrifugation at 105,000 *g* at 4°C, the supernatant was stored at 4°C. After resuspension in the same buffer, the pellets were rinsed twice and centrifuged at 800 *g* at 4°C to yield the nuclear fraction. The supernatants were added to the cytosol fraction.

Nuclear receptors were prepared according to Thorpe *et al.* (1987). The nuclear pellet was finally resuspended in 500 µl of TEMG buffer without glycerol containing 0.6 M, KCl. Nuclear receptors were extracted 1 h at 4°C under agitation. The nuclear extract was finally cleared by an ultracentrifugation at 105,000 *g* and 4°C for 1 h.

*Steroid binding assays in cytosol* were previously measured according to the French National Protocol (Martin *et al.*, 1981). An intra and interassay standard prepared from calf uterus containing known amounts of ER and PgR was included in each run.

*Elisa assays of cytosol ER and PgR* were performed as recommended by the manufacturer's package insert (Abbott ER-EIA and PgR EIA).

Nuclear receptors were measured on the same samples by enzyme immunoassay according to Thorpe *et al.* (1986).

### *Immunocytochemical detection of ER and PgR (ICC)*

Four to six µ cryostat sections were prepared from a portion of the tumours stored in liquid nitrogen. The sections were fixed for 15 min at 4°C in picric acid-paraformaldehyde, in phosphate buffer saline (PBS) pH 7.40.

ER and PgR staining was obtained by the indirect immunoperoxydase technique. The ER-ICA kit (Abbott) was used for ER, and PgR was studied as previously described by Perrot-Applanat *et al.* (1987).

A control slide was prepared for each tumour by replacing the primary antibody by normal serum of the corresponding species diluted at the same concentration of protein. Positive controls (MCF-7 cells) were also included in each series. The semi quantitative evaluation of nuclear staining was based on the intensity of staining in four classes quoted one for no staining, two for weak staining, three for intermediate staining and four for important staining, and on the percentage of positive epithelial cells.

A minimum of 200 epithelial cells were counted for each slide. Five classes of per cent positive were used, quoted one for 0%, two for less than 5% positive cells, three for 5 to 30%, four for 31 to 70% and five over 70% positive cells. The final score ranging from 1 to 20 was calculated by multiplying the grades of intensity by the percentage of positive cells (McClelland *et al.* (1986)).

Correspondence: M.F. Pichon.
Received 22 April 1991; and in revised form 14 January 1992.

*Protein determinations*

Protein assays were carried out using the bis-cinchoninic acid reagent (BCA protein assay, Pierce).

### Results

The overall results together with the clinical characteristics of the patients are listed in Table I. Our series of 15 ER(−) PgR(+) tumours can be stratified into three categories.

Patient numbers 1 and 2 received hormone therapy (contraceptive pills). In this situation, ER binding sites are partially or totally occupied, and hormone receptor complexes are less extractable by hypotonic buffers in cytosol. This results in an absence of binding sites by radioligand. However, some immunoassayable ER is found in tumour cytosol and nuclear extract. Elevated concentrations of PgR are observed in both tumours whatever the methods used.

Ten tumours (no. 3 to 12) displayed the same apparent phenotype, although none of these patients had received hormonal therapy. In all cases, immunoreactive ER is found in the nuclear fraction and in cytosol, except for tumour no. 5 in which the receptor is only present in nuclear extract.

ER was also observed in histologic sections, except for tumours no. 9 to 12. Breast cancer heterogeneity is a common feature of these tumours, and might explain that observation since PgR was present by Elisa techniques and immunocytochemistry in the same portions of the tumours.

This series of tumours may correspond to non-binding forms of ER, displaying a relatively low concentration of immunoreactive receptor in cytosol ($16.1 \pm 17.7$ fmoles mg$^{-1}$ protein), the major part of it being in the nuclear fraction ($64.2 \pm 27.3\%$ of total cellular ER). These tumours have elevated concentrations of PgR by binding and Elisa techniques, and are also positive by immunocytochemistry with high scores.

Tumour number 13 had 161 fmoles mg$^{-1}$ cytosol protein of [$^3$H] $R_{5020}$ binding protein. Using antiprogesterone receptor monoclonal antibodies, no PgR was detected in cytosol and nuclear extract. (The limit of sensitivity of the assay given by the manufacturer was 1 fmole mg$^{-1}$ protein).

The immumocytochemical assay of PgR in the tumour number 13 was also negative using different monoclonal antibodies. This result may be ascribed to the presence of a non-progesterone-receptor binding protein, perhaps the GCDFP-24 cyst protein described by Pearlman (1977).

Tumours no. 14 and 15 exhibited a particular phenotype: the oestradiol receptor was absent in the cytosol and nuclear fractions by both steorid and immunoassay on histological slides. The progesterone receptor was evidenced by all available methods. These tumours are to be considered as 'true' ER negative, PgR positive tumours.

### Discussion

The 15 tumours of that study were taken from our library of 1,211 breast cancer, among which 70 (5.78%) are ER negative-PgR positive by steroid binding assays. This phenotype has been found to occur in 3% of 1,095 primary breast cancers by Kiang and Kollander (1987) and in approximatively 6% in another study by Sarrif and Durant (1981).

This group of tumours corresponds to different situations. The first one, encountered in patients under birth control pills, or with elevated endogenous hormones, is due to receptor occupancy altering the results of standard ligand binding assays. A charcoal pretreatment of the cytosol may frequently unmask the oestradiol receptor (72% of the cases in the study of Sarrif with 18 breast tumours taken from pre or perimenopausal patients).

The same study shows that this shift in ER status is less frequent among tumours from patients over 51 years. The availability of monoclonal antibodies has provided important tools for further analysis of the ER negative PgR positive

**Table I** Clinical characteristics and receptor analysis in 15 ER(−) PgR(+) breast tumours

| *Patient No.* | *Age* | *Category of the tumour* | *Hormonal status* | *Cytosol ER (fmoles mcp)$^{-1}$ binding* | *Cytosol ER (fmoles mcp)$^{-1}$ EIA* | *Nuclear ER (fmoles mcp)$^{-1}$ EIA* | *% of total sites* | *ER score ICC* | *Cytosol PgR (fmoles mcp)$^{-1}$ binding* | *Cytosol PgR (fmoles mcp)$^{-1}$ EIA* | *Nuclear PgR (fmoles mcp)$^{-1}$ EIA* | *PgR score ICC* |
|---|---|---|---|---|---|---|---|---|---|---|---|---|
| *Patients with hormonal therapy*[a] (contraceptive pills) | | | | | | | | | | | | |
| 1 | 41 | unknown | cycling 19th d. | 0 | 13 | 62 | (60%) | 9 | 331 | 554 | 313 | 15 |
| 2 | 47 | P[a] | cycling | 0 | 18 | 24 | (35%) | 6 | 443 | 448 | 178 | 12 |
| *Patients without hormone therapy*[a] | | | | | | | | | | | | |
| 3 | 49 | P | cycling 7th d. | 0 | 32 | 94 | (57%) | 6 | 80 | 578 | 44 | 12 |
| 4 | 43 | R[b] | menopaused | $0^-$ | 11 | 61 | (57%) | 3 | 165 | 314 | 57 | 16 |
| 5 | 56 | P | menopaused | 0 | 0 | 38 | (100%) | 16 | 197 | 14 | 6 | 9 |
| 6 | 50 | R | menopaused | 0 | 8 | 53 | (72%) | 16 | 180 | 345 | 26 | 15 |
| 7 | 41 | P | cycling | 0 | 0 | 12 | (100%) | 6 | 191 | 70 | <1 | 4 |
| 8 | 57 | R | menopaused | 0 | 23 | 107 | (69%) | 9 | 156 | 44 | 3 | 16 |
| 9 | 84 | P | menopaused | 0 | 9 | 49 | (80%) | 1 | 1645 | 693 | 87 | 20 |
| 10 | 74 | P | menopaused | 0 | 58 | 6 | (12%) | 1 | 662 | 51 | 8 | 8 |
| 11 | 47 | P | cycling | 0 | 6 | 21 | (62%) | 1 | 243 | 221 | 8 | 16 |
| 12 | unknown | P | unknown | 0 | 14 | 13 | (33%) | 1 | 145 | 48 | 10 | 6 |
| 13 | 52 | P | menopaused | 0 | 0 | 0 | (0%) | 1 | 161 | 2 | 1 | 1 |
| 14 | 41 | P | cycling 16th d. | 0 | 0 | 0 | (0%) | 1 | 52 | 37 | 6 | 8 |
| 15 | 68 | P | menopaused | 0 | 0 | 0 | (0%) | 1 | 74 | 10 | 3 | 4 |

mcp = mg of cytosol protein. [a]*P* = Primitive tumour. [b]R = Relapse.

tumours, because they offer the alternative of antigenic recognition of the receptors, and are not hampered by the presence of endogenous hormones. They also allow a morphologic study of ER and PgR in breast tumours sections.

Ten tumours of our series (no. 3 to 12) have immunoreactive ER, without oestrogen binding capacity in the cytosol. The progesterone receptor is present at relatively high concentrations by binding assay (range: 80–1,645 fmoles $mg^{-1}$ cytosol protein, mean concentration 366.4 fmoles $mg^{-1}$ cytosol protein). Since PgR was measured in parallel on the same cytosol preparation, it is improbable that the negative ER results are due to a technical failure, and intra and interassay standards of ER and PgR (calf uterine powder prepared and kept in liquid nitrogen) were also included in each experiment to validate the assays.

Similar results were obtained on 9/9 tumours by Kiang *et al.*, and 7/8 tumours were found positive for ER by ICC. Our proportion of negative tumours by ICC is higher (4/10). The marked heterogeneity of breast cancer is probably the main explanation for this discrepancy. Adjacent sections for PgR immunocytochemistry were all found positive for those tumours, and positive control slides for ER and PgR were also included in the experiments.

No hormone therapy was given to those ten patients, 6/10 were menopaused, and one patient was in the follicular phase (7th day). Precise hormonal status was not available for three other patients.

A defective oestradiol receptor, with an altered steroid binding domain could be suspected to be present in these ten tumours. A similar conclusion has been drawn by Berkenstam *et al.* (1989) from their work on the hormonal regulation of ER mRNA in $T47D_{CO}$ and MCF-7 breast cancer cells. These authors showed that, in $T47D_{CO}$ cells, ER was absent by steroid binding assay and present at low concentration by Elisa assay, and that neither down regulation of ER mRNA by oestradiol, nor up regulation of PgR mRNA was observed, as it is seen in oestrogen responsive MCF-7 cells.

Finally, the most intriguing tumours are number 14 and 15. They are devoid of ER and express a functionally (at the binding level) and immunoreactive PgR. This is probably a very rare situation (2/15 = 13% of ER(−) PgR(+) in our series, or 0.17% of our overall series).

Two recent works put some insight on the molecular basis of these rare ER negative PgR positive tumours. Fuqua *et al.* (1991) have described a variant of ER devoid of exon 5 of the hormone binding domain, but able to stimulate PgR expression in three ER(−) PgR(+) breast tumours. Another situation was encountered in a T47D cell line with normal ER and an anomaly of one of the four copies of PgR gene present in these cells (Savouret *et al.*, 1991). It will thus be of interest to search for anomalies in the promoter region of the PgR gene in tumours expressing a true ER(−) PgR(+) phenotype.

### References

BERKENSTAM, A., GLAUMANN, H., MARTIN, M., GUSTAFSSON, J.A. & NORSTEDT, G. (1989). Hormonal regulation of oestrogen receptor messenger ribonucleic acid in $T47D_{CO}$ and MCF-7 breast cancer cells. *Endocrinol.*, **3**, 22.

FUQUA, S.A.W., FITZGERALD, S.D., CHAMNESS, G.C. & 5 others (1991). Variant human breast tumor estrogen receptor with constitutive transcriptional activity. *Cancer Res.*, **51**, 105.

KIANG, D.T. & KOLLANDER, R. (1987). Breast cancers negative for estrogen receptor but positive for progesterone receptor, a true entity? *J. Clin. Oncol.*, **5**, 662.

LEA, O.A., KVINNSLAND, S. & THORSEN, T. (1987). Progesterone-binding cyst protein in human breast tumour cytosol. *Cancer Res.*, **47**, 6189.

MARTIN, P.M., BRESSOT, N., DELARUE, J.C. & 4 others (1981). Protocole coopératif intercentres. In *Evaluation des moyens de diagnostic du cancer du sein.* Gest, J. (ed.), p. 263. J.M.T. Conseil: Paris.

MCCLELLAND, R.A., BERGER, U., MILLER, L.S., POWLES, T.J. & COOMBES, R.C. (1986). Immunocytochemical assay for estrogen receptor in patients with breast cancer: relationship to a biochemical assay and to outcome of therapy. *J. Clin. Oncol.*, **4**, 1171.

PEARLMAN, W.H., PENG, L.H., MAZOUJIAN, G., HAAGENSEN, D.E., WELLS, S.A. & KISLER, S.J. (1977). A specific progesterone binding component of human breast cyst fluid: its isolation and characterization. *J. Endocrinol.*, **75**, 19.

PERROT-APPLANAT, M., GROYER-PICARD, M.T., LORENZO, F. & 5 others (1987). Immunocytochemical study with monoclonal antibodies to progesterone receptor in human breast tumours. *Cancer Res.*, **47**, 2652.

SAVOURET, J.F., FRIDLANSKY, F., ATGER, M., MISRAHI, M., BERGER, R. & MILGROM, E. (1991). Origin of the high constitutive level of progesterone receptor in T47-D breast cancer cells. *Mol. C. Endo.*, **75**, 157.

SARRIF, A.M. & DURANT, J.R. (1981). Evidence that estrogen-receptor-negative, progesterone-receptor-positive breast and ovarian carcinomas contain estrogen receptor. *Cancer*, **48**, 1215.

THORPE, S., LYKKESFELDT, A.E., VINTERBY, A. & LONSDORFER, M. (1986). Quantitative immunological detection of estrogen receptors in nuclear pellets from human breast cancer biopsies. *Cancer Res.* (suppl.), **46**, 4251s.